# "Refusal to Attend Chapel": The Home's Reply

**Published:** 1917-10-06

**Authors:** 


*To the Editor of* The Hospital.

### "Refusal to Attend Chapel": The Home's Reply.

*To the Editor of* The Hospital.

Sir,—My attention has been drawn to a paragraph appearing in the current number of The Hospital concerning the treatment of certain patients at St. Anne's Home, Bridlington. May I point out a few inaccuracies? The dispute did not concern Sunday services, but the daily morning and evening prayer of the Home. I enclose a copy of the rules, from which you will see that we allow perfect freedom to patients of any creed on Sundays to attend whatever place of worship they please, but we do expect our patients to attend prayers on week-days if such attendance does not prejudice their health.

The dispute occurred at 9 p.m., the hour at which the lights are extinguished in the smoking and recreation rooms of the Home; therefore there is not much point in the patients' charge that "they were treated like naughty children and sent to bed." I do not think, Sir, that you, with your clinical experience, will blame us heavily for our observance of the maxim "Early to bed" in the case of patients committed to our care.

I admit that we did refuse to keep these particular patients. The dispute took place on Saturday night. We gave them the whole of Sunday to think the matter over. When Monday morning came, and they still refused to abide by our rules, we paid their fares home, so that they might go to other Homes which have no rules.

The men were sent to us by various Pensions Committees. Under Rule 9 (page 2) you will see by the sum charged that the payments would not cover the cost of maintenance at present prices. No conditions were suggested to us by the Pensions Committees of exceptional treatment. We sent copies of our rules to the committees, and we imagined that the patients they sent would abide by them.

We try to do our best for all the patients who enter the Home. The fact that a chapel in the Home affords an opportunity for the worship of God has never appeared to be a drawback in the eyes of the Council, which exists to carry out the wishes of the founders.—I am your obedient servant,

Alexander Macdonald of the Isles.

September 25, 1917. Vice-Chairman of the Council.

[There is an engaging candour in Sir Alexander Macdonald's courteous letter which makes a note on his defence of his committee's action a matter of courtesy too. Rule 3 of the Home deserves to be quoted. It runs thus :

"3. St. Anne's Home is a Church of England Institution, but charity knows no distinction of persons, and St. Anne's is open to members of all creeds and denominations. Perfect freedom is allowed as regards Sunday services. All patients are at liberty to attend whatever place of worship they please, and no hindrance is put in the way of the fullest religious freedom of every inmate. At the same time the rule of attending morning and evening prayers is expected to be observed by all who are inmates of the Home, unless they are prevented by illness."

According to the letter of the law, then, the authorities of St. Anne's Home were justified in what they did. But, with all respect to their rule and to the wishes of the founders, the rule itself is inexpedient, and likely, when infringed, to create the prejudice which has in fact been excited. For it is illogical to open a Home to members of all denominations while yet insisting on attendance at "prayers." Undenominational religious teaching is unworkable. That, we fear, is the blunt truth. We hope that the Lady Superior, who also wrote to us, will take this as an answer to her letter also. We believe that she will agree that her own contribution does not add anything material to Sir Alexander Macdonald's longer statement, and we have not room for both.—Ed. The Hospital.]

